# A Method for Prediction the Trajectory of Table Tennis in Multirotation State Based on Binocular Vision

**DOI:** 10.1155/2022/8274202

**Published:** 2022-04-14

**Authors:** Guan Lan Cai

**Affiliations:** Sports and Public Art Department, Zhengzhou University of Aeronautics, Henan, Zhengzhou 450015, China

## Abstract

The research on the space trajectory of high-speed moving and flying objects has very important research significance and application value in the fields of sports, military, aerospace, and industry. Table tennis has the characteristics of small size, fast flight speed, and complex motion model. It is very suitable as an experimental object for the study of flying object trajectory. This study takes table tennis as the research object to carry out research on the trajectory prediction of flying objects and builds a trajectory prediction system based on the trajectory prediction model, combining the constraints of the simple physical motion model and the deviation correction of the double LSTM neural network. Aiming at the problem of trajectory extraction of flying table tennis balls, a high-speed industrial camera was used to build a table tennis trajectory extraction system based on binocular vision. A multicamera information fusion method based on dynamic weights is proposed for the prediction of the trajectory of flying table tennis. In order to solve the problems that some model parameters are difficult to measure and the model is too complicated in the traditional physical motion model of table tennis trajectory, a method combining simple physics is proposed. This paper proposes a trajectory prediction model with motion model constraints and dual LSTM neural network bias correction. Experiments show that the proposed method can greatly improve the accuracy of the trajectory extraction and prediction system and can achieve a certain success rate of hitting.

## 1. Introduction

The research on the space trajectory of high-speed moving and flying objects has attracted the attention of many researchers, and it has great research significance and application value in the fields of sports, military, aerospace, and industry. In the field of sports, the live broadcast of ball games can model and predict the trajectory of the ball on the field in real time to help the audience better experience the game, and offline can also help athletes better target training in the military field. In the field of aerospace [1–3], the tracking and interception of missiles, the prediction of the movement and landing of artillery shells, and the trajectory planning of unmanned fighter jets all have great national defense value; in the aerospace field, spacecraft launch and return, satellite orbits around the Earth, etc., need to track high-speed aircraft. Research: in the industrial field, the use of robots to realize the grasping of dynamic target objects has gradually become a new application hotspot [4–6].

The research on the trajectory of moving and flying objects mainly needs to solve the two major problems of trajectory tracking and trajectory prediction, of which trajectory tracking is the premise of trajectory prediction. At present, the main technical means to realize the trajectory tracking of flying objects are radar, laser, and vision. Among them, visual positioning has the characteristics of strong flexibility, high precision, and many applications [5]. It is very suitable for the research of flying object trajectories. The main research content is the use of visual positioning. The system extracts the complete trajectory of the flying ball and performs trajectory prediction, which includes research difficulties such as target recognition, 3D reconstruction, information fusion, system real-time synchronization, trajectory tracking, and trajectory prediction. Table tennis has the characteristics of small size, fast-flying speed, and complex motion model, which is very suitable for the experimental object standard of flying ball trajectory prediction [6–10].

Binocular vision is an important branch of computer vision and a hotspot of current robotics research. Binocular vision imitates the way the human eye processes information and obtains three-dimensional information of objects from two-dimensional images. Specifically, it is to shoot the same three-dimensional object from two different viewpoints, obtain two-dimensional images from two different perspectives, and then use the principle of optical triangulation to obtain a disparity map, thereby obtaining the three-dimensional information of the object [11–13]. Compared with other methods (such as holography and lens plate 3D imaging), binocular vision can directly imitate the way the human eye processes information, which is more reliable and convenient. The research content of binocular vision is very extensive, mainly including image acquisition, camera calibration, feature extraction, stereo matching, and three-dimensional reconstruction, which can be applied to many fields. The research on the trajectory modeling of table tennis is actually tracking and predicting the trajectory of table tennis so that the robot can know the information of the landing position of the table tennis ball in advance and prepare for hitting the ball. In layman's terms, this study is to imitate the function of human eyes, design a visual system of a table tennis robot, and transmit various motion information of table tennis to a PC for processing [14–16].

According to the difference in the number of cameras used, the vision system can be divided into monocular vision, binocular vision, and follicular vision. Monocular vision refers to using only one camera to shoot, so its structure is relatively simple, and the calibration process is relatively easy, but the monocular vision system has strict requirements on the experimental environment, and the viewing angle is relatively narrow. Although the visual vision system has high recognition accuracy and a wide field of view, due to the increase in the number of cameras, the resulting calculation amount increases, the image processing work is more heavy, and it is difficult to meet the original intention of the real-time robot vision system. Therefore, at present, most researchers mainly adopt the binocular vision system, which is to observe the same object through two viewpoints, which is similar to the human visual system. Limited by monocular vision, only one camera can be used [17–20]. The shadow of the ball is very important for the three-dimensional positioning of the ball. Therefore, the monocular system has strict requirements on the lighting conditions and the flight speed and range of the ball, and it cannot adapt to the trajectory of the table tennis ball in complex environments. The binocular vision simulates the human eyes using two cameras for image acquisition. Due to the difference in the poses and orientations of the two cameras, the three-dimensional positioning of the space objects is carried out through the parallax of the pixels of the same name mapped on the imaging planes of the two cameras and the principle of geometric triangulation. Compared with monocular vision, binocular vision can not only directly obtain depth information without considering too many environmental factors but also has a larger detection range. At present, many table tennis trajectory studies are based on a binocular vision system for table tennis trajectory extraction [21–23].

At present, the research on the trajectory prediction of table tennis is mainly divided into prediction based on the traditional physical motion model and prediction based on the machine learning model. The trajectory prediction of table tennis based on the traditional physical motion model needs to consider various complex physical field factors, such as gravity, Magnus force, air resistance, ball rebound force, and landing friction, which will undoubtedly bring about the high performance of the model: complexity and low robustness. In order to solve the above deficiencies, Zhao proposed a motion model. First, the *K*-means algorithm was used to cluster the motion trajectories, and then, Fourier series were used to fit them to obtain an extended continuous motion model [24–26]. On the basis of ECMM, a new motion state estimation method based on an expectation-maximization algorithm is proposed to make the motion trajectory more accurately predicted. At the same time, the category in ECMM is regarded as a latent variable, and the likelihood of the motion state is expressed as the difference between the Gaussian mixture model ballistic prediction and observation of the system. In recent years, machine learning has also gradually moved towards table tennis research. Wang proposed a trajectory prediction algorithm for rotating table tennis balls based on the Kalman filter. First, dynamic modeling of the rotation curve, including topspin, backspin, left-spin, and combined spin, was used to optimize the curve using the Kalman filter. For simulating and predicting the flight trajectory of the ball, the error between the final reconstructed curve and the real curve of the model is 2.42%, indicating that the model has high accuracy and effectiveness [27–29]. Lu proposed an adaptive measurement covariance discrete Kalman trajectory estimation algorithm to solve the error problem caused by factors such as high-speed motion-blurred images of table tennis, air resistance, and camera imaging distortion. The algorithm achieves accurate tracking of the target motion trajectory by dynamically adjusting the size of the measurement covariance, which further lays a foundation for the prediction of table tennis and the hitting arm. When the moving speed is greater than 5 m/s, the algorithm can effectively overcome the interference of measurement noise and data loss and achieve a good tracking effect. Sebastian proposes a deep conditional generative model for trajectory prediction in view of the fact that Ping-Pong trajectories belong to time-series data. A neural network maps full or partial trajectories to a Gaussian-distributed latent space and back, allowing fast inference of future values of trajectories based on previous observations [30].

The main research goal of this study is to analyze and model the movement trajectory of table tennis based on binocular vision. The premise of trajectory modeling is to realize the detection and tracking of moving table tennis. The specific implementation process is as follows: using target detection technology, the motion area of the table tennis ball in flight is separated, and the center coordinates of the table tennis ball are obtained; then, the camera calibration technology combined with its imaging model is used to locate the target in three dimensions, and the flight trajectory of the table tennis ball is tracked according to the target tracking technology to obtain the table tennis ball; and finally, combined with the trajectory model of the table tennis ball, the trajectory prediction of the flying table tennis ball is realized. The main content of this study is based on the binocular vision system, target recognition, three-dimensional positioning and tracking of fast-moving table tennis balls, physical modeling of their motion trajectory, and algorithm implementation to ensure accurate follow-up experiments. Predict the trajectory and landing of a table tennis ball.

The sections of this paper are arranged as follows. First, the research background and research significance are given, and then, the research status of the moving object detection technology, moving object tracking technology, and trajectory prediction technology involved in this study is briefly introduced.  Section 2 first introduces the basic principle of binocular vision, then introduces the hardware and software architecture of the binocular vision system, and finally describes the key technical principles in the binocular vision system in detail.  Section 3 studies, in detail, the initial stage of the table tennis trajectory, which is also a very important stage, that is, the target detection stage, and proposes a new method, which integrates color segmentation, background subtraction, and ellipse fitting, and complements each other's advantages. To solve the “smear” problem caused by the high-speed flying table tennis balls, this method can accurately obtain the two-dimensional position of the ball center.  Section 4 studies the three-dimensional positioning of the target center point and the tracking of the motion trajectory and completes the three-dimensional positioning of the table tennis ball.  Section 5 is summary and outlook.

## 2. Research on Binocular Vision

### 2.1. Composition of Binocular Vision System

**Figure 1:**
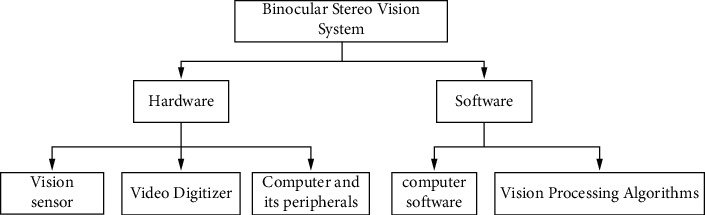
Binocular stereo vision software and hardware structure.

The binocular stereo vision system is a system composed of two viewpoints, including hardware and software. Firstly, select the appropriate hardware equipment according to the task requirements of the vision system and design the program structure of the software part to form the ontology vision system of the humanoid robot. The hardware part includes visual sensors, video digitizing equipment, computers, and peripheral devices; the software part includes computer software and visual processing algorithms. The specific structure diagram is shown in Figure 1.

**Figure 2:**
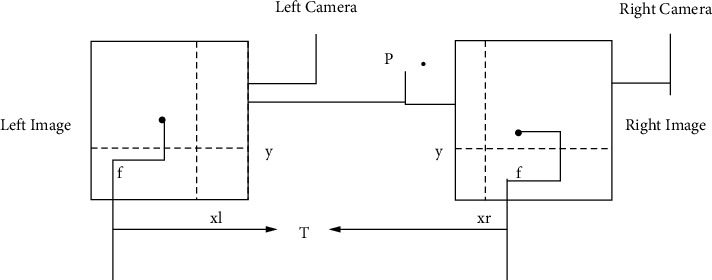
Schematic diagram of binocular stereo imaging.

Binocular vision is a technology based on the principle of the human eye parallax.  Figure 2 shows its imaging principle.

Figures 1 and 2 show the model-building process of the dual-target stereo image, which is the basic model of the entire research process and the data processing center of the experimental case. In Figure 2, T is the baseline distance, that is, the distance between the projection centers of the left and right cameras, and *f* is the camera focal length. There is a point *P* in the three-dimensional coordinate system, and its position coordinates are (*x*_*P*_, *y*_*P*_, *z*_*P*_). It is assumed that the optical axes of the left and right cameras are perfectly aligned, the parameters of the two cameras are the same, and the lens has no distortion. Point *P* will have two imaging points in the left and right images, and their abscissas are *x*_*l*_ and *x*_*r*_, respectively. Since the two cameras are arranged in parallel, the ordinates of the imaging points are equal. According to the similar triangle relationship, it can be deduced as

(1) $$\begin{array}{c} \left\{\begin{array}{c} x_{l}=f\frac{X_{p}}{Z_{p}},\\ x_{r}=f\frac{X_{p}-T}{Z_{p}},\\ y=f\frac{Y_{p}}{Z_{p}}. \end{array}\right. \end{array}$$

Arranging formula (1), we can get the three-dimensional coordinates of point *P* in the camera coordinate system as the following equation:

(2) $$\begin{array}{c} \left\{\begin{array}{c} Xp=\frac{T\cdot x_{l}}{x_{l}-x_{r}},\\ Yp=\frac{T\cdot y}{x_{l}-x_{r}},\\ Zp=\frac{T\cdot f}{x_{l}-x_{r}}. \end{array}\right. \end{array}$$

**Figure 3:**
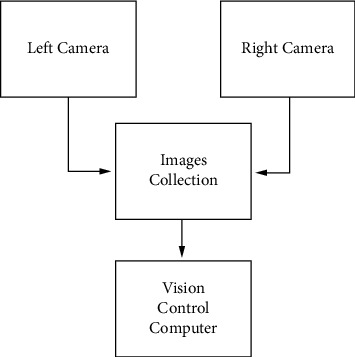
Vision system hardware structure.

The hardware structure of binocular vision is shown in Figure 3. Two cameras are used to collect video signals, and then, the cameras are connected to the control machine through the image acquisition card, and the analog signals, collected by the acquisition equipment, are converted through sampling, filtering, quantization, and other steps for image data output to the computer.

The selection of the camera has a great influence on the experiment, so it is necessary to select a suitable camera based on various factors. The following points should be considered when choosing a camera.

#### 2.1.1. Frame rate

Real-time performance and accuracy are the two main goals pursued by the binocular vision system. For a table tennis ball with a ball speed of 5 m/s and a diameter of 40 mm (international standard ball), when the exposure time is 40 mm/5 m/s = 8  ms, the table tennis ball will move 40  mm within the exposure time, and the two-dimensional image will show the phenomenon of “smearing” occurs, which will cause a large error in the identification and positioning results of the table tennis ball; the table tennis ball, with a speed of 5 m/s, lasts about 600  ms from one side of the table to the other side to obtain as many consecutive times as possible in this process. For pictures, the higher the rate of the camera, the better the frame.

#### 2.1.2. Color

Since the color of the object contains rich feature information, this study is implemented in a specific color space, so a color camera is selected.

#### 2.1.3. Pixel

The larger the pixel value of the camera, the higher the discrimination accuracy, but the increase in the amount of transmitted data leads to a longer transmission time, which in turn leads to a smaller frame rate. Therefore, it is necessary to select the pixel value as large as possible under the condition that the frame rate is satisfied.

#### 2.1.4. Transmission interface

The size of the transmission rate has a great influence on the real-time performance of the system and also affects the frame rate of the camera. Similarly, on the premise of satisfying the frame rate of the camera, select a camera with a faster transmission rate. Considering the above factors, this study selects the Pike F-032 B/C camera of Microvision Company, and its specific parameters are shown in Table 1.

**Figure 4:**
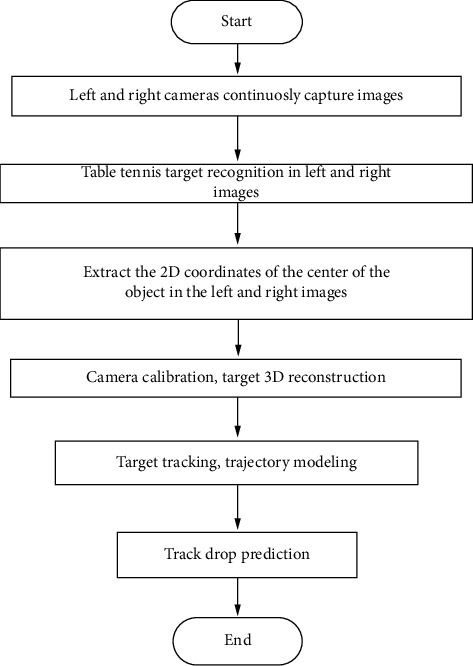
Vision system software structure.

The software part of the system is implemented in the Visual Studio 2008 development environment combined with the OpenCV visual library, as shown in Figure 4. The left and right cameras carry out continuous image acquisition through internal development, obtain continuous table tennis flying pictures, use the target recognition method to detect the area where the table tennis ball is located in the picture, and then obtain the center coordinates of this area, when the left and right projection images are processed. After the corresponding image coordinates are obtained, the three-dimensional position of the table tennis ball can be reconstructed and calculated, and the future position of the table tennis ball can also be predicted according to the historical position information of the table tennis ball to realize.

### 2.2. Introduction of Key Technologies in Binocular Vision

The key technologies in the binocular vision system are as follows.

#### 2.2.1. Image Acquisition

The acquisition method of binocular vision is binocular imaging, that is, two collectors take images of the same scene from two viewpoints, use the same collector to take images of the same scene from the two viewpoints successively, or use the same one. The collector relies on an optical imaging system to acquire both images. From a geometrical point of view, the image acquisition process is a process of spatial transformation of the scene in the three-dimensional objective world through projection, that is, the 3D objective scene is converted into a 2D image plane through projection; from an optical point of view, it is a process of converting the three-dimensional objective:

(3) $$\begin{array}{c} \left\{\begin{array}{c} u=\frac{x}{\text{d}x}+u_{0},\\ v=\frac{y}{\text{d}y}+v_{0}. \end{array}\right. \end{array}$$

The process of converting the light radiation intensity in the scene into the grayscale of the image as (3) can also be defined as follows:

(4) $$\begin{array}{c} \left[\begin{array}{c} u\\ v\\ 1 \end{array}\right]=\left[\begin{array}{ccc} \frac{1}{dx} & 0 & u_{0}\\ 0 & \frac{1}{dy} & v_{0}\\ 0 & 0 & 1 \end{array}\right]\left[\begin{array}{c} x\\ y\\ 1 \end{array}\right]. \end{array}$$

The image acquisition device needs to receive the external stimulus, generate the corresponding analog electrical signal, and then convert it (the electrical signal) into a digital form so that it can be used by the computer. Commonly used acquisition devices include CCD cameras, CMOS cameras, CID frequency acquisition cards, and scanners. The basic performance indicators of image acquisition equipment are linear response, sensitivity, signal-to-noise ratio, unevenness, shutter speed, and transmission rate. The vision system software structure is shown in Figure 4.

#### 2.2.2. Camera Calibration

Camera calibration is the calculation process of obtaining camera parameters through a set of known reference points (pixel points in a two-dimensional image). In binocular stereo vision, it is expressed as follows. By calculating the corresponding pixel points in the two 2D images, the camera's own parameters and attitude parameters are determined, so as to obtain the corresponding relationship between the object points in the 3D world coordinates and the imaging points in the 2D image coordinates. This determines the three-dimensional position information of the object. At present, the camera calibration methods can be roughly divided into traditional calibration technology, camera self-calibration technology, and active vision calibration technology. When the traditional calibration technology is calibrated, the camera completes the calibration by finding the feature primitives with known coordinates on a specific reference object. It is a method that directly uses a mathematical transformation to calculate the internal and external parameters of the camera model; the camera self-calibration technology is more flexible, does not rely on a specific reference object, and calculates the camera model by comparing the corresponding relationship between the images in the environment. This technology has strong adaptability to the surrounding environment, but compared with traditional calibration technology, its accuracy is still very different. Active vision calibration technology is currently only applicable to scenes with known camera motion or controllable camera motion, which can be solved linearly. The accuracy of camera calibration has a great influence on the accuracy of subsequent target tracking and trajectory prediction and is an important research topic in the vision system:

(5) $$\begin{array}{c} {\text{Focus}}_{t+1}={\text{Focus}}_{t}+T_{t+1}, \end{array}$$

where Focus is the value of *t* + 1 focus and *T* is the overall focus in the period *t*+1; the equation means that the focus of current equals to the potential focal of the former time and the current ones.

#### 2.2.3. Image Preprocessing and Feature Extraction

In order to more effectively extract the information in the two-dimensional image obtained by image acquisition, it is necessary to perform certain preprocessing and processing on the original image. On the one hand, in the process of image acquisition, due to the influence of noise, the problem of geometric distortion may be caused. In order to restore the corresponding relationship between the three-dimensional object and the two-dimensional image, image coordinate transformation (translation, rotation, scaling, etc.) is required. On the other hand, improve the visual quality of the image, adjust the image amplitude, and improve the image clarity. After image preprocessing, the extraction and analysis of image features and computer processing will be more convenient. Image preprocessing techniques include image enhancement, denoising, enhancement of edge features, histogram correction, and spatial filtering. In order to describe the position of the camera, the world coordinate system (*x*_*W*_, *y*_*W*_, *z*_*W*_) has been introduced. The camera coordinates can be converted into the world coordinate system, and the conversion relationship between the two is shown in following equation:

(6) $$\begin{array}{c} \left[\begin{array}{c} X_{C}\\ Y_{C}\\ Z_{C}\\ 1 \end{array}\right]=\left[\begin{array}{cc} R & t\\ 0^{T} & 1 \end{array}\right]\left[\begin{array}{c} X_{W}\\ Y_{W}\\ Z_{W}\\ 1 \end{array}\right]. \end{array}$$

#### 2.2.4. Image Correction

The goal of image correction is to standardize the distribution of the epipolar lines in the geometric constraints of the epipolar and turn them into parallel lines. It only needs to scan the image to match the feature points without the calculation of the epipolar lines, which simplifies the calculation and improves the matching efficiency:

(7) $$\begin{array}{c} H_{I}=-\int_{0}^{1}P_{I}\left(i\right)\log\;P_{I}\left(i\right)\text{d}i, \end{array}$$

where *P*_*I*_ (*i*) represents the probability density when the pixel gray value is *i*.

#### 2.2.5. Stereo Matching

Stereo matching is the most important and difficult part of binocular vision. The difference between the stereo imaging images is not caused by the change and movement of the scene itself, but is caused by the difference in the angle of view when shooting, so that many factors in the scene are integrated into the gray value of the image, such as lighting, object shape information, noise interference, and image distortion. Therefore, it is very difficult to accurately match images containing so many interference factors:

(8) $$\begin{array}{c} MI_{I1,I2}=H_{I1}+H_{I2}-H_{I1,I2}, \end{array}$$

where *I*1 and *I*2 represent two images, respectively, and *H*_*I*1_ and _*I*2_ are joint descendants, and their formula is as follows:

(9) $$\begin{array}{c} H_{I1,I2}=-\int_{0}^{1}\int_{0}^{1}P_{i1\text{,}i2}\left(i1\text{,}i2\right)\log\;P_{i1,i2}\left(i1,i2\right)\text{d}\left(i1\right)\text{d}\left(i2\right), \end{array}$$

where *P*_*i*1,*i*2_ represents the joint probability density, which is defined as follows:

(10) $$\begin{array}{c} P_{i1\text{,}i2}\left(i,k\right)=\frac{1}{n}\sum_{P}T\left[\left(i,k\right)=\left(I_{1p},I_{2p}\right)\right], \end{array}$$

where *i* and *k* are, respectively, the gray value of the pixel points of the two images, *n* is the total number of pixels, *P* is the pixel point, and *T* operation represents the random distribution of the corresponding gray level.

To improve the efficiency of matching, three problems need to be solved: the accuracy of feature extraction, the search for the essential attributes between features, and the ability to correctly match the selected stable algorithms for features. The matching primitives used in the matching algorithm mainly include two things: one is to extract measurement descriptors from all image pixels, including pixel gray values, local area gray functions, and convolution image symbols; the other is image features, including contour points, edges, and corners. According to the different primitives, stereo matching algorithms can be divided into regional stereo matching algorithms, feature-based stereo matching algorithms, and phase-based stereo matching algorithms. According to the different optimization theoretical methods used, stereo matching algorithms can be divided into local stereo matching algorithm and global stereo matching algorithm.

The purpose of 3D reconstruction is to redraw the original spatial structure of the object through the 2D scene image. After the camera's imaging geometric model and matching relationship are obtained through camera calibration and matching, 3D reconstruction can be performed. After calculating the three-dimensional coordinates of several points in the scene, the reconstructed object is displayed by using the three-dimensional display technology of the computer. In the reconstruction process, the factors that affect the accuracy are the digital effect of image acquisition, the error caused by camera calibration, the accuracy of feature extraction, and the accuracy of matching.

## 3. Research on Motion Trajectory Prediction

This section introduces the methods used to predict the trajectory of the target object. First, the recurrent neural network is introduced. This neural network is a network with “memory.” Using the recurrent neural network to predict the time-series model has a good effect. This section focuses on an efficient and accurate recurrent neural network that uses long- and short-term memory to predict the trajectory. Since in visual measurement, the measurement accuracy in the depth direction is much lower than other directions, the smaller the *Z*_*w*_ obtained when processing the three-dimensional coordinates of the target obtained by multiple sets of binocular vision, the higher the confidence. Aiming at the problem of information fusion across multiple groups of visual systems, this study proposes an information fusion strategy. According to the principle of the smaller *Z*_*w*_ and the larger weight coefficient, the weights of different groups of stereo vision systems *Z*_*w*_ are dynamically adjusted:

(11) $$\begin{array}{c} \xi_{1}=\frac{Z_{2}+Z_{3}}{2\left(Z_{1}+Z_{2}+Z_{3}\right)},\\ \xi_{2}=\frac{Z_{1}+Z_{3}}{2\left(Z_{1}+Z_{2}+Z_{3}\right)},\\ \xi_{3}=\frac{Z_{2}+Z_{1}}{2\left(Z_{1}+Z_{2}+Z_{3}\right)}. \end{array}$$

The above formulas are, respectively, the coordinate value weight coefficients of the three groups of stereo vision systems, and Z1, Z2, and Z3 are, respectively, the *Z*-axis coordinate values extracted by the three groups of stereo vision. When there is a stereo vision system that does not detect the extraction coordinates of the target, the corresponding value is 0. The three-dimensional coordinate value extracted by the entire multieye vision can be obtained from the above weight coefficient:

(12) $$\begin{array}{c} \left[\begin{array}{c} XW\\ YW\\ ZW \end{array}\right]=\left[\begin{array}{ccc} \xi 1 & \xi 2 & \xi 3 \end{array}\right]\left[\begin{array}{ccc} X1 & Y1 & Z1\\ X2 & Y2 & Z2\\ X3 & Y3 & Z3 \end{array}\right]. \end{array}$$

Since the designed trajectory extraction system uses multiple cameras to collect stereo image pairs, when the cameras are out of sync, the stereo image pairs on the timestamp will cause trajectory extraction errors, and the errors will gradually expand and affect the final trajectory prediction accuracy. Therefore, it is necessary to design an appropriate camera synchronization trigger method so that the trajectory extraction system can take into account the accuracy and real-time performance. To simplify the experiment, this study does not consider the fluctuation of the camera frame rate. Since the hardware synchronous triggering method can ensure that different cameras can shoot synchronously, this study designs a hardware synchronous triggering device in the trajectory extraction system, which is connected to the camera to synchronously control the acquisition of stereo images.

**Figure 5:**
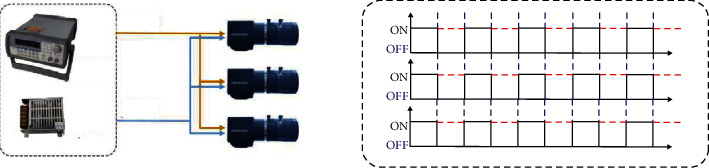
Hardware trigger structure.

**Figure 6:**
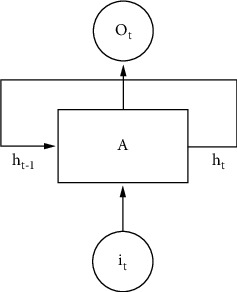
Schematic diagram of the structure of the recurrent neural network.

Figure 5 shows a schematic diagram of the external hardware triggering of multivision. It can be seen from Figure 5 that the 12-power generator is used to supply power to the camera, and the signal generator is used to issue a square wave trigger signal to trigger multicamera synchronization. In this experiment, the camera is connected to the computer through the industrial camera trigger line, and the interface is USB3.0. After testing, the experimental equipment can transmit an 80 Hz video stream stably, so the trigger frequency of the signal generator is set to 80 Hz, that is, the multicamera system uses an 80 Hz frequency for simultaneous image acquisition. A schematic diagram of the structure of the recurrent neural network is shown in Figure 6.

Recurrent neural network is another efficient deep learning algorithm different from convolutional neural network. Using recurrent neural networks to solve time-series prediction problems can get more accurate prediction results. Unlike the previous convolutional neural network used for image recognition, the recurrent neural network is a neural network with “memory.” The output of the convolutional neural network is only related to the input this time and has nothing to do with the previous input. The recurrent neural network is different; it will remember the previous input and use the previous input to affect the output this time. Taking advantage of this feature, recurrent neural networks can be effectively used to process and predict sequence data. Figure 6 is a schematic diagram of the structure of a recurrent neural network. As can be seen from Figure 6, the output of the main structure A is determined by the input at the current moment and the hidden state at the previous moment, and a new hidden state is also generated. The main structure is unchanged at different times, so the recurrent neural network can also be regarded as infinite replication at different time periods.

## 4. Trajectory Prediction

Due to the low accuracy of the binocular camera used in the test, in order to facilitate the measurement, this design allows the target object to move similar to a pendulum within the range that the binocular camera can capture. Through the MobileNet-SSD model, the pixel coordinates of the target object during the moving process can be obtained, and 30 sets of three-dimensional coordinates can be obtained through three-dimensional reconstruction. Some of the coordinates are shown in Table 2.

The hyperparameter set before using long- and short-term memory prediction are shown in Table 3.

**Figure 7:**
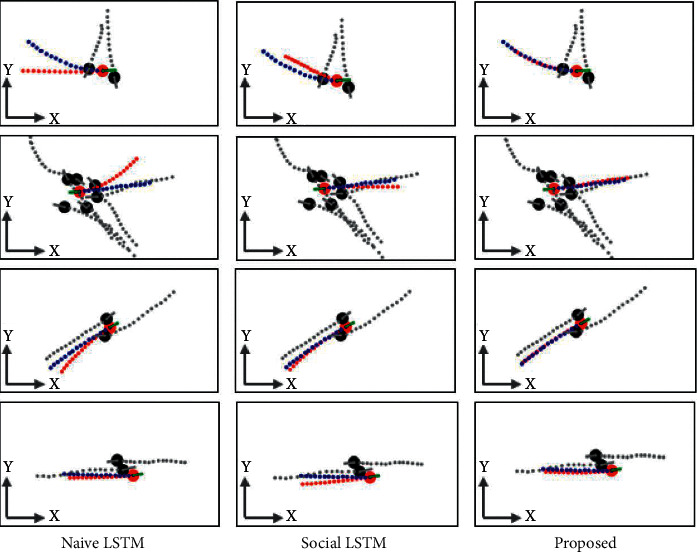
Prediction of the drop of table tennis.

After the model training is completed, 20 sets of data are input for verification, and the first 11 sets of three-dimensional coordinates are set to predict the next number. In this way, the coordinates in each direction will get 7 predicted values. The true and predicted values of the *x*-axis and *y*-axis are shown in Figure 7.

This study completes the three-dimensional positioning and trajectory tracking of flying table tennis. In the three-dimensional positioning stage, the various coordinate systems used in the vision system and their interrelationships are introduced. The camera is calibrated using the calibration program that comes with OpenCV, and the internal and external parameters of the camera are obtained. Combined with the work of the previous two sections, the Ping-Pong 3D positioning of the sphere. The accuracy of 3D positioning is verified by experiments. The experimental results show that the standard error is less than 20 mm, which meets the requirements of trajectory prediction. In the trajectory prediction stage, the research status of domestic table tennis trajectory modeling is introduced in detail, and the force in the flight process of table tennis is analyzed. It is implemented algorithmically.

## 5. Conclusion

The binocular vision-based recognition and tracking system designed in this study uses the calibration target to calibrate the binocular camera to obtain the internal and external parameters, then uses the stereo correction to make the two optical axes of the binocular camera parallel, and then performs feature matching to obtain the disparity map. Finally, the three-dimensional coordinates of the target object are obtained through parallax. Through the position coordinates of the target object in the previous frames of images, the trajectory prediction algorithm is used to predict the position coordinates of the target object in the next frame to complete the detection and tracking of the target object. The test results show that the accuracy of target object detection and trajectory prediction can reach the expected expectations. According to the requirements, the design is mainly divided into three parts: the binocular vision part, the target detection part, and the trajectory prediction part. The design of this study has done a lot of research on existing algorithms in terms of object recognition and trajectory prediction and made some improvements. The model has also been tested with ideal results. However, the training of deep models, especially the tuning of parameters, is a very difficult task. The long process requires a long-term accumulation of knowledge and laboratory support. Due to my limited level, in the process of target recognition and detection, it is found that when the target is far away from the binocular camera or the input image is too large, the recognition accuracy rate is very low, which is also a common problem in China. In the field of target detection, the SSD algorithm needs to be further optimized and further research is needed.

## Figures and Tables

**Table 1:** Pike F-032 performance parameter table.

Transmission interface	IEEE 1394b-800 M/s
Maximum frame rate	208 fps
Color	Multicolor
Resolution	640 *∗* 480
Sensor	Kodak KAI -0340
Sensor type	1/3 CCD progressive
RAM	64 MB

**Table 2:** Partial 3D position coordinates.

*X*	*Y*	*Z*
−10.6698	−7.5569	−54.1610
−10.5289	−7.7478	−54.1178
−11.5002	−7.9651	−54.5648
−10.9345	−7.8654	−53.8727
−10.5416	−7.91197	−54.4278
−8.1061	−6.0426	−54.5678

**Table 3:** Long short-term memory network hyperparameter settings.

Hidden nodes	Layers	Series length	Batch	Steps	Groups' data
50	3	15	20	10000	20

## Data Availability

The data used to support the findings of this study are available from the corresponding author upon request.
